# Behavior Changes for Smokers and Betel Quid Chewers Participating in the Organized Oral Mucosal Screening Between 2010 and 2021 in Taiwan

**DOI:** 10.3390/cancers17030397

**Published:** 2025-01-25

**Authors:** Pattaranan Munpolsri, Chiu-Wen Su, Sam Li-Sheng Chen, Amy Ming-Fang Yen

**Affiliations:** 1 School of Dentistry, College of Oral Medicine, Taipei Medical University, Taipei 110, Taiwan; pattaranan_nut@hotmail.com; 2 Department of Internal Medicine, National Taiwan University Hospital, Taipei 100, Taiwan; hfn202@gmail.com; 3 School of Oral Hygiene, College of Oral Medicine, Taipei Medical University, 250 Wu-Hsin Street, Taipei 110, Taiwan; samchen@tmu.edu.tw

**Keywords:** smoking, betel nut, areca, behavior modification, screening, cancer

## Abstract

Objective: To investigate the likelihood of smoking and betel quid chewing behavior changes, as well as the associated factors, among subjects in a nationwide oral cancer screening program in Taiwan, where oral cancer is the fourth leading malignancy in men. Methods: The Taiwanese Oral Cancer Screening Program targets smokers and betel quid chewers over the age of 30. Between 2010 and 2021, 2,578,245 individuals participated in the program at least twice. During each visit, the central data monitoring center collected information on gender, age, education level, smoking and betel quid chewing habits, the quantities consumed, and diagnoses of oral potentially malignant disorders (OPMDs) and oral cancer. A three-state Markov chain exponential regression model was employed to assess the probabilities of behavior change—deterioration, maintenance, or improvement—and the factors influencing these changes. Results: The largest proportion of both smokers and betel quid chewers remained unchanged in the repeated attendees. The smoking cessation transition result was less than 10%, whereas the reduction in dosage exceeded 20%. However, the discontinuation of betel quid chewing was observed in more than 20% of individuals in the low-dose group, and there was also a reduction in dosage of more than 20%. The positive OPMDs caused the progression of the Net Force Progression (NFP) from an initial stage of none, as well as the NFP between low and high doses of both smoking and betel quid chewing. Higher levels of education, residing in urban areas, and receiving screenings in large hospitals help to avoid the progressive NFP between low and high doses of smoking. Additionally, these factors also help to prevent the development of the NFP from an initial stage of none, as well as the progressive NFP between low and high doses of betel quid chewing. Conclusions: Participation in an oral cancer screening program can effectively enhance hazardous oral behaviors, including smoking and betel quid chewing. Although it may not have a substantial effect on smoking cessation, it can decrease the daily consumption of cigarettes. This approach demonstrates encouraging outcomes in encouraging individuals to quit betel quid chewing and decrease their daily consumption. Higher levels of education, urban residency, and receiving screenings at large hospitals all have a good impact on reducing dangerous oral cancer behaviors.

## Simple Summary

This study examines changes in oral risk behaviors, including smoking and betel quid chewing, following participation in the Taiwanese Oral Cancer Screening Program. It also explores factors associated with these behavioral changes. The findings highlight that participation in the oral cancer screening program is an essential intervention that helps individuals reduce and quit oral risk behaviors, particularly among urban residents and those with at least a high school education. This can lead to long-term benefits for oral health and a reduced risk of oral cancer in the future. To enhance the program’s effectiveness, efforts should focus on targeting individuals living in rural areas and those with limited educational opportunities.

## 1. Introduction

In the year 2020, there were more than 370,000 new cases of oral cancer that were detected, and the disease was responsible for more than 170,000 fatalities worldwide [1,2,3]. Oral cancer screening programs are an essential alternative for reducing the death rate associated with oral cancer. Both Sankaranarayanan (2013) and Rajaraman (2015) agree with the assertion that oral cancer screening programs contribute to a decrease in the mortality rate associated with oral cancer [4,5]. According to Chuang (2017), there is a significant reduction in the death rate of oral cancer in stages III and IV, with a relative risk of 0.53 [6]. Smoking cigarettes, chewing betel quid, drinking alcohol, oral infections, and oral potentially malignant disorders (OPMDs) are the leading causes of oral cancer [7,8,9,10]. Oral potentially malignant disorders (OPMDs) are a group of conditions in the mouth that have a higher risk of developing into oral cancer, including leukoplakia, erythroplakia, oral submucous fibrosis, lichen planus, and actinic cheilitis. These disorders manifest as various types of lesions or abnormalities in the oral mucosa and serve as early warning signs for potential oral cancer, particularly squamous cell carcinoma. Common risk factors for OPMDs include tobacco use, alcohol consumption, human papillomavirus (HPV) infection, and a diet low in fruits and vegetables. Regular monitoring, biopsy, and histopathological examination are essential for managing these disorders and intervening early if malignant transformation occurs [11,12,13,14]. This screening program is designed not only to facilitate the early detection of oral cancer but also to educate participants about its associated risk factors and behaviors. While considerable evidence underscores the benefits of early diagnosis and treatment, there is a paucity of research focusing on how such screening initiatives influence participants’ health behaviors. This study seeks to explore the extent to which individuals alter their smoking and betel quid chewing habits and to examine the demographic and personal characteristics—such as gender, age, education, and health status—that may affect their capacity for behavioral modification.

The analysis of projected oral behavior changes across different severity levels, along with the identification of associated factors, can help to develop coping strategies and improve screening programs. This information would be valuable for the participants, especially for those exhibiting the most adverse behavior patterns.

## 2. Materials and Methods

### 2.1. Study Design

This is a retrospective cohort study that uses data from the Oral Cancer Screening Program in Taiwan.

### 2.2. Data Source and Collection

This study utilized data from the Taiwan Oral Cancer Screening Program spanning 2010–2021. In addition to detecting early-stage oral cancer for timely treatment, the screening program also provided education on risk factors and methods for maintaining good oral health. Information on demographic characteristics and behaviors—such as sex, age, education level, living area, cigarette consumption, and betel quid chewing—was collected through face-to-face interviews conducted in communities and hospitals. A structured questionnaire was used for this purpose. Participants underwent visual examinations of their oral cavity, performed by trained dentists or physicians, to detect Oral Potentially Malignant Disorders (OPMDs), including clinical diagnoses such as oral leukoplakia, erythroleukoplakia, erythroplakia, oral submucous fibrosis, and verrucous hyperplasia [6]. The program was integrated with the National Cancer Registry to confirm OPMD diagnoses. Screening results were documented using International Classification of Diseases (ICD) codes, with Chuang (2017) providing detailed descriptions of this screening program and coding [6]. It is acknowledged that data collection through interviews is susceptible to biases, including interviewer bias, social desirability bias, and recall bias. To mitigate these biases, all of the interviews were conducted by trained professionals or personnel proficient in interview techniques.

### 2.3. Sample Size and Variables

**Figure 1:**
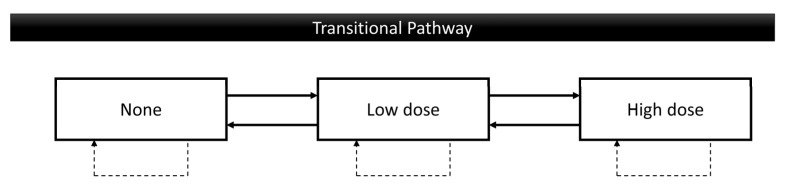
Possible behavior change pathways of smoking and betel quid chewing. None: never or cessation; Low dose: using less than 20 values per day; High dose: using 20 and higher values per day.

This study includes all 5.4 million participants from the Taiwan Oral Cancer Screening Program between 2010 and 2021. Participants younger than 30 years old and those who attended screening only once were excluded, leaving 2.57 million individuals for analysis. The age restriction to over 30 years was applied because previous research indicated that individuals younger than 30 are not at significant risk for oral cancer [15]. The probability of behavior changes at each stage is presented as percentages to analyze patterns of behavioral changes. Factors related to these changes were analyzed using variables such as age, sex, education level, living area, screening place, and OPMD results. The main focus of this study is on possible behavior changes, which are categorized into the following pathways: unchanged, none to low dose, none to high dose, low dose to none, low dose to high dose, high dose to none, and high dose to low dose. These three levels of behavior for both smoking and betel quid chewing are defined as follows: “none” refers to never using or cessation, “low dose” refers to using less than 20 units per day, and “high dose” refers to using more than 20 units per day (Figure 1). Age was divided into the following two groups: younger (≤60 years) and elder (60+ years). Education level was classified into low and high, where individuals with the highest education level of elementary or middle school were categorized as low, and those with high school or college education were categorized as high. Screening places were grouped into large hospitals (medical centers and regional hospitals) and small hospitals (local hospitals and clinics). OPMD results were classified into positive and negative findings, with individuals diagnosed with leukoplakia, erythroleukoplakia, erythroplakia, oral submucous fibrosis, or verrucous hyperplasia categorized as having positive findings.

### 2.4. Statistical Analysis

**Figure 2:**
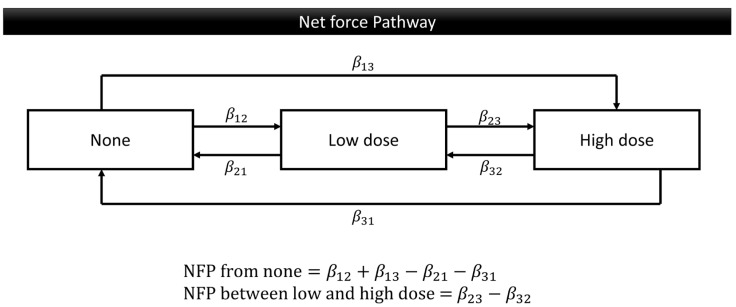
The Net Force Progression of oral risk behavior, as determined by covariance. There are the following two patterns: the NFP from none and the NFP between low and high doses.

The demographics of this data, including the gender, age group, education level, living area, screening place, OPMDs, and oral cancer, were summarized as frequency and percent. The Markov chain model provides an investigation of the transitional probability of deteriorating, maintaining, or improving. Using the exponential regression form representing behavior weights for respective progression and regression, we translated three transition parameters into the following two patterns of Net Force Progression (NFP): the NFP from none and the NFP between low and high doses. The NFP from none was calculated by subtracting any regressions to the none stage from progressions from the none stage. This was performed to analyze the tendency to initiate oral risky behavior compared to ceasing such behavior. The NFP between the low and high doses was determined by subtracting the regressions from the high to low dose from the progressions from the low to high dose so to study the trends in increasing or decreasing the daily dosage of oral risky behaviors (Figure 2). This study categorized missing data as one level of the data, allowing for adjustments during the analysis. The investigation on transitional probabilities and associated factors for behavior change was conducted using Markov chain Monte Carlo (MCMC) estimation. All of the analyses were performed using SAS version 9.4 (SAS Institute, Cary, NC, USA).

## 3. Results

### 3.1. Characteristics of Participants

Table 1 provides a comprehensive summary of the proportions of smoking and betel quid chewing behaviors among participants across different demographic categories at their first screening. The highest proportion for each demographic characteristic was observed in low-dose smoking, except for the positive finding of OPMDs, which showed the highest proportion in high-dose smoking (42.8%). For betel quid chewing, the highest proportion across all demographic categories was observed in non-chewers.

### 3.2. Transitional Probability of Oral Risk Behaviors

**Figure 3:**
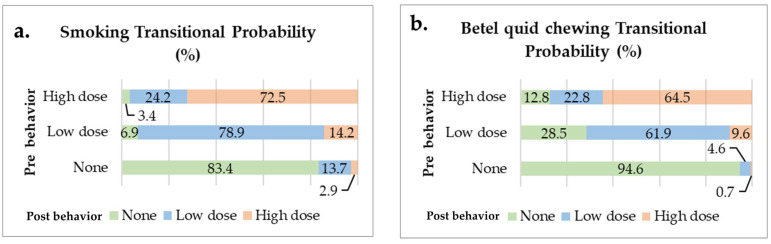
Transitional probability for each behavior type (**a**) of smoking and (**b**) betel quid chewing.

The Markov chain model demonstrates the probability of behavior change after participating in the oral cancer screening program for the groups categorized as none, low dose, and high dose. Most participants did not change their behavior. However, when considering the low-dose stage, which is the intermediate stage, it was found that participants were more likely to worsen their behavior (moving to the high-dose category) rather than quit smoking, with probabilities of 14.2% and 6.9%, respectively. On the other hand, the low-dose betel quid chewing group showed a higher likelihood of quitting chewing (none) rather than increasing their consumption (high dose), with probabilities of 28.5% and 9.6%, respectively. Furthermore, when comparing the high-dose betel quid chewing group to the high-dose smoking group, it was found that the betel quid chewing group was nearly four times more likely to quit chewing than the smoking group was to quit smoking (Figure 3).

### 3.3. Effect of Risk Factors on Multiple Transitions for Smoking Behavior

**Figure 4:**
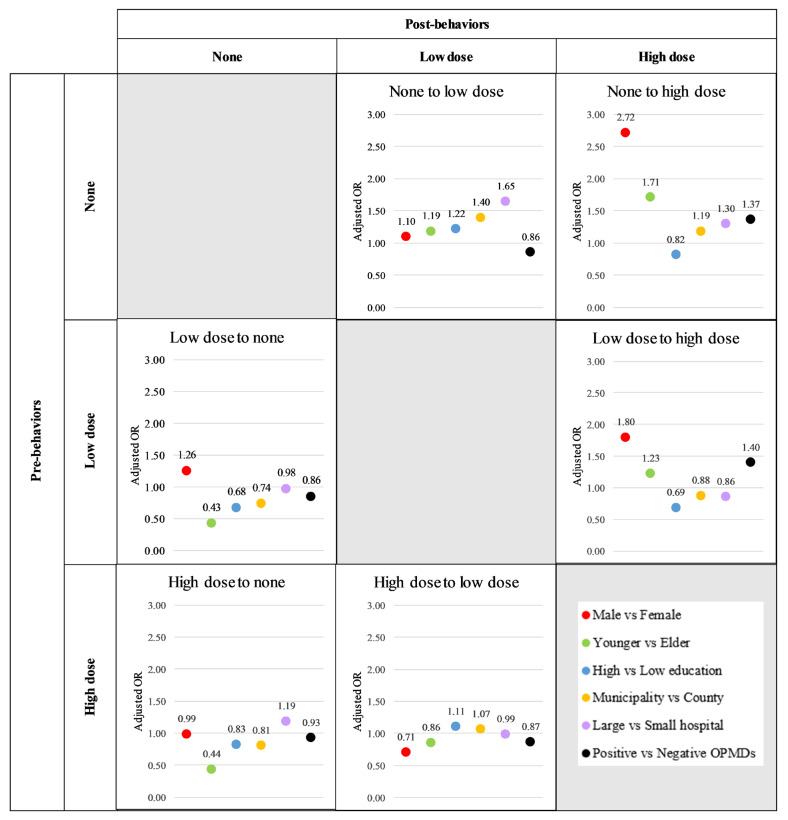
Associated factors of smoking behavior transformations.

Table 2 presents the adjusted relative risk (aRR) and 95% confidence interval (95%CI) for the effect of each factor on the Net Force Progression (NFP) of smoking, accounting for all risk factors. The results indicate that, overall, no factor significantly inhibited the initiation of smoking (the NFP from the none stage). However, the stage-specific analysis revealed that a positive finding of OPMDs significantly reduced the likelihood of initiating low-dose smoking, with an aRR (95%CI) of 0.86 (0.84–0.87). Additionally, a high level of education was associated with a reduced likelihood of initiating high-dose smoking, with an aRR (95%CI) of 0.82 (0.80–0.85) (Figure 4; Supplementary Table S1).

For the NFP between low and high doses, factors such as a high level of education, living in a municipal area, and screening at a large hospital significantly inhibited the increase in the smoking dose, with respective aRR (95%CI) values of 0.62 (0.62–0.63), 0.83 (0.82–0.84), and 0.87 (0.86–0.88). Notably, when examining the stage-specific effects, screening at a large hospital not only inhibited the increase in the smoking dose, with an aRR (95%CI) of 0.86 (0.86–0.87), but also encouraged the participants to reduce their smoking volume, as reflected by an aRR (95%CI) of 1.19 (1.16–1.21) (Figure 4; Supplementary Table S1).

### 3.4. Effect of Risk Factors on Multiple Transitions for Betel Quid Chewing Behavior

Table 3 presents the adjusted relative risk (aRR) and 95% confidence interval (95%CI) for the effect of various factors on the Net Force Progression (NFP) of betel quid chewing, accounting for all risk factors. The results indicate that only a high level of education, living in a municipal area, and screening at a large hospital were significantly associated with inhibiting the initiation of betel quid chewing, with respective aRR (95%CI) values of 0.35 (0.34–0.36), 0.28 (0.27–0.29), and 0.62 (0.60–0.64).

For the NFP between low and high doses, both a high level of education and living in a municipality significantly inhibited the increase in the betel quid chewing dose, with respective aRR (95%CI) values of 0.62 (0.61–0.64) and 0.85 (0.83–0.87). While screening at a large hospital appeared to reduce the likelihood of dose escalation, this effect was not statistically significant.

**Figure 5:**
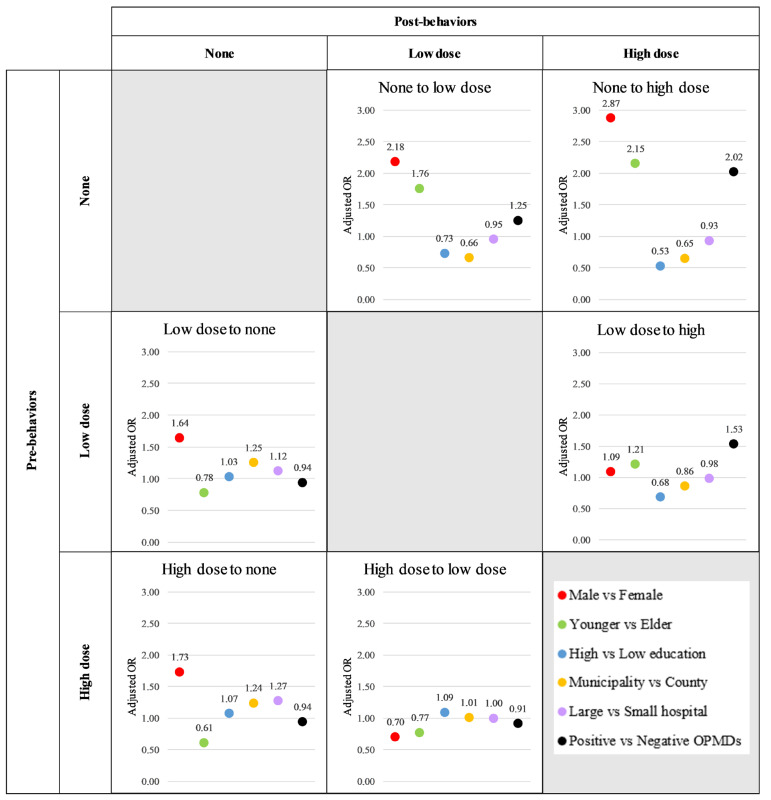
Associated factors of betel quid chewing behavior transformations.

However, when considering the stage-specific effects, individuals with a high level of education were significantly more likely to reduce their betel quid chewing dose, at an aRR (95%CI) 1.07 (1.04–1.1) (Figure 5; Supplementary Table S2).

## 4. Discussion

This study shows that most people do not change their behavior. It found that smoking cessation occurs in less than 10% of cases, while the cessation of betel quid chewing occurs in more than 10%. In the group that starts with low-dose smoking, there is a higher rate of progressive transition than cessation. Conversely, in the group that starts with low-dose betel quid chewing, cessation is more common than progressive transition. Considering the factors influencing behavior change can enhance our understanding and promote future behavior improvement.

### 4.1. Smoking

This study demonstrated that individuals diagnosed with OPMDs are more inclined to encourage smoking initiation among non-smokers rather than assisting current smokers in their efforts to quit. However, an extensive analysis was conducted on individuals who only smoked. The results revealed that positive OPMDs (Oral Potentially Malignant Disorders) only led to smoking cessation in the high-dose group (Supplementary Table S3). A study conducted in Korea also found that illness could significantly induce smoking improvement, with adjusted odds ratios (aORs) of 1.4 (95%CI: 1.07–1.85) for hypertension and 1.68 (95%CI: 1.03–2.75) for cardiovascular disease [16]. However, cancer was found to non-significantly induce smoking improvement, with an aOR of 4.63 (95%CI: 0.9–23.93) [16]. These data indicate that people are willing to adjust their behavior when they begin to realize they are in a dangerous situation. The relatively low response to OPMDs may be due to an insufficient awareness and understanding of the dangers associated with this disease.

This study showed that males are more inclined to encourage smoking initiation among non-smokers rather than assisting current smokers in their efforts to quit. Previous studies did not find that males influence smoking behavior improvement, with non-significant adjusted odds ratios (95%CI) of 0.96 (0.78–1.17) for the United States [17] and 0.81 (0.43–1.5) for China [18]. It is possible that men, particularly in Asian societies, often hold the belief that they must assume the role of family leader. This role may increase stress due to the burden of responsibilities, potentially contributing to smoking as a means of relaxation. However, cultural values and practices vary significantly across geographical regions, so further research is necessary to examine the impacts of stress in greater detail in the future.

This study demonstrated that younger individuals are more likely to encourage non-smokers to start smoking rather than aiding smokers in quitting. However, previous studies conducted in the US, China, and Korea found that age did not significantly impact smoking behavior modification [17,18,19]. The adjusted odds ratios (95%CI) for smoking cessation were 1.19 (0.96–1.47) for younger individuals in the US [17], 0.94 (0.4–2.23) for the elderly in China [18], and 2.35 (0.48–11.45) for the elderly in Korea [19].

This study demonstrated that individuals with a higher education are more likely to encourage non-smokers to start smoking rather than helping current smokers quit. However, higher education can also promote a reduction in the smoking dosage. Previous studies have shown that higher education significantly influences smoking behavior modification, with adjusted odds ratios (95%CI) of 3.19 (1.02–9.98) in Korea [19] and 0.54 (0.4–0.71) in Pakistan for smoking prevalence [20]. Considering the results of this study, it is suggested that the increased difficulty associated with higher education may lead individuals to rely on smoking to alleviate stress. Nonetheless, higher educational attainment can also enhance the awareness and understanding of the dangers of smoking, thereby encouraging individuals to reduce and control their smoking levels.

This study demonstrated that urban living is more likely to encourage non-smokers to start smoking rather than aiding current smokers in quitting. However, urban living can also promote a reduction in the smoking dosage. Supporting this, a study in Pakistan found that residing in urban areas induced smoking spread, with an adjusted odds ratio (95%CI) of 1.55 (1.28–1.87) [20]. Considering the results of this study, it is suggested that living in urban areas, where people often face higher stress levels, intense competition, and greater pollution than in rural areas, leads to increased smoking initiation. Nevertheless, urban residents have better access to medical centers and educational resources compared to those in rural areas. This access allows individuals to become more aware of the dangers of smoking and to control their smoking levels, even if they do not quit entirely.

This study demonstrated that screening in large hospitals is more likely to encourage non-smokers to start smoking rather than aiding current smokers in quitting. However, screening in large hospitals can also promote a reduction in the smoking dosage. Large hospitals or medical centers are hubs of numerous health experts, and their advice is crucial in influencing individuals to modify their behavior and pay more attention to their health [21,22,23,24,25,26]. While complete smoking cessation may not be achieved, the influence of these healthcare professionals can significantly reduce the daily smoking dosage. Therefore, the distribution of healthcare centers, particularly large-scale centers, along with efforts to enhance their credibility—such as advertising expert physicians or modern technology—could potentially encourage greater behavioral improvements. Additionally, future studies should also consider the convenience of participation in such programs as an important factor.

### 4.2. Betel Quid Chewing

This study provides evidence that individuals diagnosed with OPMDs are more inclined to initiate the behavior of chewing betel quid rather than discontinue it if they are already users. Moreover, the diagnosis also tends to enhance their everyday consumption. Prior studies have established a correlation between betel quid consumption and the likelihood of developing OPMDs. Nevertheless, there was a lack of research on the impact of an OPMD diagnosis on betel quid chewing behavior.

According to this study, men who have never chewed betel quid are more inclined to start doing so rather than stop if they are already using it. Additionally, males tend to increase their daily consumption of betel quid. Previous research has shown that both males and females influence the progression of betel quid use [27,28,29,30]. The study provided the adjusted hazard rate ratio (aHRR) and 95% confidence interval (95%CI) for women in Malaysia as an aHRR (95%CI) of 5 (4.2–6) [30] and, for males in Taiwan, as 1.38 (1.2–1.59) [28]. The study reported the adjusted odds ratio (aOR) and 95% confidence interval (CI) for women in Bangladesh as an aOR (95%CI) of 1.2 (1.04–1.37) [29], and, for males in Taiwan, as 1.14 (1.01–1.3) [27]. The evidence suggests that both males and females can contribute to the progress of betel quid chewing, with varying impacts according to the geographical region.

This study reveals that persons below the age of 60 are more likely to initiate betel quid consumption than discontinue it if they are already users. Moreover, the diagnosis leads to an increase in their daily chewing frequency. Taiwan conducted a study that revealed a lower likelihood of older adults joining the betel quid chewing group compared to younger individuals. The study reported an adjusted odds ratio (aOR) of 0.84 (with a 95% confidence interval (95%CI) of 0.72–0.97) [27]. Nevertheless, in Malaysia, as individuals aged, their chances of quitting betel quid chewing declined [30]. The adjusted hazard rate ratio (aHRR) and 95% confidence interval (CI) support this, with values of 0.18 (0.05–0.63) for individuals aged 41–50 years and 0.11 (0.03–0.38) for those aged 51 years and above [30]. An adjusted odds ratio (aOR) of 63.54 (95% confidence interval: 43.02–96.82) from a study in Bangladesh showed that older people significantly influenced the prevalence of betel quid usage [29]. Differences in health awareness and adherence to traditional practices can explain the variability in health outcomes across regions.

This study reveals that those with higher levels of education demonstrate an enhancement in their betel quid chewing behaviors, such as the cessation or reduction in the amount consumed. Existing research provided evidence that higher levels of education were associated with improved betel quid chewing behaviors. We observed that, in Bangladesh, individuals with a high level of education significantly prevented betel quid usage, with an adjusted odds ratio (aOR) of 0.17 and a 95% confidence interval (95%CI) ranging from 0.15 to 0.2 [29]. In Taiwan, individuals with lower levels of education were less likely to attempt quitting betel quid, with an adjusted odds ratio (aOR) of 0.58 (95% confidence interval [CI]: 0.34–0.98) [31]. They also had a higher prevalence of betel quid chewing, with an aOR of 2.02 (95%CI: 1.75–2.34) [27]. Furthermore, individuals with a junior high school education had an adjusted hazard rate ratio (aHRR) of 2.33 (95%CI: 1.83–2.97), while those with a senior high school education had an aHRR of 2.51 (95%CI: 1.99–3.17) compared to college and above [28]. Increased levels of education can help to improve the comprehension of the hazards associated with chewing betel quid.

This study observed that individuals living in urban areas have positive changes in their betel quid chewing behaviors, such as stopping or reducing the amount consumed. A Bangladeshi study found that living in cities was linked to a lower likelihood of using betel quid. The study’s adjusted odds ratio (aOR) was 0.58, and its 95% confidence interval (95%CI) was from 0.54 to 0.62 [29]. Urban areas exhibit dynamic trends, making it easier to replace traditional betel quid chewing practices than in other areas.

This study discovered that implementing screening programs in large hospitals has a substantial positive impact on betel quid chewing behaviors, specifically in motivating individuals to quit, when compared to screening programs in small hospitals. Hospitals or large medical centers are centers of specialized health specialists, which means that the advice and knowledge from these sources are highly reputable and have a greater chance of effectively influencing people’s actions [21,22,23,24,25,26]. However, considering the ease of access to service areas remains essential for future studies.

### 4.3. Limitation

This study focused on a specific group of individuals who participated in the screening program on at least two occasions. Therefore, it is not appropriate to extrapolate the findings of this study to individuals who only took part in the program once. Furthermore, future studies should consider environmental factors, such as the accessibility of betel quid and cigarettes, the availability of hospitals, the convenience of program participation, and stress-related factors. However, due to geographical and cultural differences, the results of studies conducted in other countries may differ from the findings of this study.

## 5. Conclusions

Participation in an oral cancer screening program contributes to enhancing smoking and betel quid chewing behaviors. While the rates of smoking cessation were below 10% for both low- and high-dose smokers, the high-dose group showed a significant drop of over 20% in daily smoking, which is worth mentioning. Furthermore, participation in this program benefited betel quid chewing behavior, as the low-dose group achieved a cessation rate of over 20% and the high-dose group achieved a reduction in dosage of over 20%. Furthermore, those with higher levels of education, residing in urban areas, and receiving screening at large hospitals contributed to the enhancement of oral risk behaviors. For better oral health and to reduce the risk of oral cancer, encouraging the public to reduce risky oral behaviors is essential. Participating in oral cancer screening programs is one way to help people monitor their oral health and to enhance their knowledge and understanding of proper oral care practices. Special emphasis can be placed on targeting groups residing in rural or suburban areas with education levels below a high school degree.

## Figures and Tables

**Table 1:** Distribution of smoking and betel quid chewing status by participant demographics at the first screening.

	Smoking			Betel Quid Chewing			Total (n)
	None (%)	Low Dose (%)	High Dose (%)	None (%)	Low Dose (%)	High Dose (%)	
Gender							
Male	20.6	47.7	31.7	75.1	17.7	7.3	2,190,949
Female	32.7	52.5	14.8	85.7	10.9	3.4	387,296
Age							
≤60 years	16.6	51.8	31.6	74.3	18.2	7.5	1,877,590
61+ years	38	39.2	22.8	83	12.5	4.5	700,655
Education							
Elementary and middle school (Low education)	29.3	40.9	29.8	74.8	16.9	8.3	515,778
High school and college (High education)	17.5	54.5	28	79.9	15	5.1	860,054
unknown	22.9	47.2	29.8	75.2	17.7	7.1	1,202,413
Living area							
Urban	18.3	52	29.7	80.8	14	5.2	1,609,524
Rural	29.2	42.5	28.3	69.9	21	9.1	968,718
Screening place							
Large hospital	22.3	49.2	28.5	78.2	15.5	6.2	726,200
Small hospital	22.4	48.1	29.5	76.1	17.1	6.8	1,851,831
Unknown	9.3	65.9	24.8	82.2	15	2.8	214
OPMD screening results							
Positive	15	42.2	42.8	59.8	23.9	16.3	193,256
Negative	23	48.9	28.1	78	16.1	5.9	2,384,989
Overall	22.4	48.4	29.2	76.7	16.7	6.7	2,578,245

**Table 2:** Associated factors for the Net Force Progression of smoking behavior progression.

	Net Force Progression from None Stage						Net Force Progression Between Low and High Dose					
	RR	95%CI		aRR	95%CI		RR	95%CI		aRR	95%CI	
Gender												
Male	1.94	1.85	2.06	2.4	2.26	2.57	2.51	2.48	2.55	2.53	2.49	2.56
Female	ref			ref.			ref			ref		
Age												
Younger (≤60 years)	13.74	13.42	14.06	10.85	10.53	11.17	1.22	1.21	1.23	1.43	1.42	1.45
Elder (60+ years)	ref			ref			ref			ref		
Education												
Low education	ref			ref			ref			ref		
High education	5.31	5.12	5.5	1.8	1.74	1.86	0.68	0.67	0.69	0.63	0.62	0.63
Living area												
Municipality	3.51	3.42	3.6	2.77	2.7	2.84	0.78	0.78	0.79	0.83	0.82	0.84
County	ref			ref			ref			ref		
Hospital												
Small hospital	ref			ref			ref			ref		
Large hospital	2.06	2.01	2.12	1.86	1.8	1.91	0.88	0.87	0.89	0.87	0.86	0.88
OPMD result												
Positive	1.67	1.6	1.74	1.46	1.4	1.53	1.74	1.72	1.77	1.61	1.59	1.63
Negative	ref			ref			ref			ref		

**Table 3:** Associated factors for the Net Force Progression of betel quid chewing behavior progression.

	Net Force Progression from None Stage						Net Force Progression Between Low and High Dose					
	RR	95%CI		aRR	95%CI		RR	95%CI		aRR	95%CI	
Gender												
Male	2.34	2.19	2.46	2.21	2.09	2.37	1.54	1.5	1.59	1.57	1.53	1.62
Female	ref			ref			ref			ref		
Age												
Younger (≤60 years)	4.4	4.26	4.56	7.93	7.66	8.2	1.48	1.44	1.51	1.58	1.54	1.62
Elder (60+ years)	ref			ref			ref			ref		
Education												
Low education	ref			ref			ref			ref		
High education	0.49	0.48	0.51	0.35	0.34	0.36	0.74	0.72	0.76	0.62	0.61	0.64
Living area												
Municipality	0.26	0.25	0.26	0.28	0.27	0.29	0.92	0.9	0.94	0.85	0.83	0.87
County	ref			ref			ref			ref		
Hospital												
Small hospital	ref			ref			ref			ref		
Large hospital	0.61	0.59	0.63	0.62	0.6	0.64	1	0.98	1.02	0.98	0.95	1
OPMD result												
Positive	3.24	3.11	3.37	2.87	2.76	2.99	1.73	1.68	1.77	1.68	1.63	1.72
Negative	ref			ref			ref			ref		

## Data Availability

The original contributions presented in this study are included in the article/Supplementary Materials. Further inquiries can be directed to the corresponding author(s).

## Supplementary Materials

The following supporting information can be downloaded at: https://www.mdpi.com/article/10.3390/cancers17030397/s1, Table S1: Associated factors of smoking behavior transformations [aRR(95%CI)]; Table S2: Associated factors of betel quid chewing behavior transformations [aRR(95%CI)]; Table S3: Associated factors of only smoking behavior transformations [aRR(95%CI)].

## Abbreviation

The following abbreviation is used in this manuscript:

OPMDs	Oral Potentially Malignant Disorders

## References

[1] World Cancer Research Fund International (2022). Mouth and Oral Cancer Statistics.

[2] WHO (2023). Oral Health.

[3] Sung H., Ferlay J., Siegel R.L., Laversanne M., Soerjomataram I., Jemal A., Bray F. (2021). Global Cancer Statistics 2020: GLOBOCAN Estimates of Incidence and Mortality Worldwide for 36 Cancers in 185 Countries. CA Cancer J. Clin..

[4] Sankaranarayanan R., Ramadas K., Thara S., Muwonge R., Thomas G., Anju G., Mathew B. (2013). Long term effect of visual screening on oral cancer incidence and mortality in a randomized trial in Kerala, India. Oral Oncol..

[5] Rajaraman P., Anderson B.O., Basu P., Belinson J.L., Cruz A.D., Dhillon P.K., Gupta P., Jawahar T.S., Joshi N., Kailash U. (2015). Recommendations for screening and early detection of common cancers in India. Lancet Oncol..

[6] Chuang S.L., Su W.W.Y., Chen S.L.S., Yen A.M.F., Wang C.P., Fann J.C.Y., Chiu S.Y.H., Lee Y.C., Chiu H.M., Chang D.C. (2017). Population-based screening program for reducing oral cancer mortality in 2,334,299 Taiwanese cigarette smokers and/or betel quid chewers. Cancer.

[7] Anwar N., Pervez S., Chundriger Q., Awan S., Moatter T., Ali T.S. (2020). Oral cancer: Clinicopathological features and associated risk factors in a high risk population presenting to a major tertiary care center in Pakistan. PLoS ONE.

[8] Xiao X., Wang Z., Zhou X., Zhang Z. (2021). Chapter 1—Oral Cancer. Pharynx-Diagnosis and Treatment.

[9] Jose M., Rajagopal V., Thankam F.G., Sharma C.P. (2021). Chapter 9—Oral Tissue Regeneration: Current Status and Future Perspectives. Regenerated Organs.

[10] Senevirathna K., Pradeep R., Jayasinghe Y.A., Jayawickrama S.M., Illeperuma R., Warnakulasuriya S., Jayasinghe R.D. (2023). Carcinogenic Effects of Areca Nut and Its Metabolites: A Review of the Experimental Evidence. Clin. Pract..

[11] Hübbers C.U., Akgül B. (2015). HPV and cancer of the oral cavity. Virulence.

[12] American Dental Association (2024). HPV and Oral Cancer.

[13] Sathish N., Wang X., Yuan Y. (2014). Human Papillomavirus (HPV)-associated Oral Cancers and Treatment Strategies. J. Dent. Res..

[14] Lechner M., Liu J., Masterson L., Fenton T.R. (2022). HPV-associated oropharyngeal cancer: Epidemiology, molecular biology and clinical management. Nat. Rev. Clin. Oncol..

[15] Chuang S.L., Wang C.P., Chen M.K., Su W.W.Y., Su C.W., Chen S.L.S., Chiu S.Y.H., Fann J.C.Y., Yen A.M.F. (2018). Malignant transformation to oral cancer by subtype of oral potentially malignant disorder: A prospective cohort study of Taiwanese nationwide oral cancer screening program. Oral Oncol..

[16] Eum Y.H., Kim H.J., Bak S., Lee S.H., Kim J., Park S.H., Hwang S.E., Oh B. (2022). Factors related to the success of smoking cessation: A retrospective cohort study in Korea. Tob. Induc. Dis..

[17] Fagan P., Augustson E., Backinger C.L., O’connell M.E., Vollinger R.E., Kaufman A., Gibson J.T. (2007). Quit attempts and intention to quit cigarette smoking among young adults in the United States. Am. J. Public Health.

[18] Feng G., Jiang Y., Li Q., Yong H.-H., Elton-Marshall T., Yang J., Li L., Sansone N., Fong G.T. (2010). Individual-level factors associated with intentions to quit smoking among adult smokers in six cities of China: Findings from the ITC China Survey. Tob. Control.

[19] Yeom H., Lim H.S., Min J., Lee S., Park Y.H. (2018). Factors affecting smoking cessation success of heavy smokers registered in the intensive care smoking cessation camp (data from the National Tobacco Control Center). Osong Public Health Res. Perspect..

[20] Nadeem M., Malik M.I., Ullah A., Junaid N. (2024). Smoking Dynamics: Factors Supplementing Tobacco Smoking in Pakistan. IEEE Trans. Comput. Soc. Syst..

[21] Stead L.F., Buitrago D., Preciado N., Sanchez G., Hartmann-Boyce J., Lancaster T. (2013). Physician advice for smoking cessation. Cochrane Database Syst. Rev..

[22] Carlebach S., Hamilton S. (2009). Understanding the nurse’s role in smoking cessation. Br. J. Nurs..

[23] Rice V.H., Hartmann-Boyce J., Stead L.F. (2013). Nursing interventions for smoking cessation. Cochrane Database Syst. Rev..

[24] Luh D.-L., Chen S.L.-S., Yen A.M.-F., Chiu S.Y.-H., Fann C.-Y., Chen H.-H. (2016). Effectiveness of advice from physician and nurse on smoking cessation stage in Taiwanese male smokers attending a community-based integrated screening program. Tob. Induc. Dis..

[25] Tammemägi M.C., Berg C.D., Riley T.L., Cunningham C.R., Taylor K.L. (2014). Impact of lung cancer screening results on smoking cessation. J. Natl. Cancer Inst..

[26] Siewchaisakul P., Luh D.-L., Chiu S.Y.H., Yen A.M.F., Chen C.-D., Chen H.-H. (2020). Smoking cessation advice from healthcare professionals helps those in the contemplation and preparation stage: An application with transtheoretical model underpinning in a community-based program. Tob. Induc. Dis..

[27] Lin C.-F., Wang J.-D., Chen P.-H., Chang S.-J., Yang Y.-H., Ko Y.-C. (2006). Predictors of betel quid chewing behavior and cessation patterns in Taiwan aborigines. BMC Public Health.

[28] Yap S.-F., Ho P.-S., Kuo H.-C., Yang Y.-H. (2008). Comparing factors affecting commencement and cessation of betel quid chewing behavior in Taiwanese adults. BMC Public Health.

[29] Flora M.S., Mascie-Taylor N., Rahman M. (2012). Betel quid chewing and its risk factors in Bangladeshi adults. WHO South-East Asia J. Public Health.

[30] Ghani W.M., Razak I.A., Yang Y.-H., Talib N.A., Ikeda N., Axell T., Gupta P.C., Handa Y., Abdullah N., Zain R.B. (2011). Factors affecting commencement and cessation of betel quid chewing behaviour in Malaysian adults. BMC Public Health.

[31] Lai C.-S., Shieh T.-Y., Yang Y.-H.C., Chong M.-Y., Hung H.-C., Tsai C.-C. (2006). Factors associated with quitting areca (betel) quid chewing. Community Dent. Oral Epidemiol..

