# Supplementary material for: Behavior Changes for Smokers and Betel Quid Chewers Participating in the Organized Oral Mucosal Screening Between 2010 and 2021 in Taiwan
Source: Cancers (Basel). 2025 Jan 25;17(3):397. doi: 10.3390/cancers17030397 (PMC11816278; doi:10.3390/cancers17030397)
Supplement: Supplementary file 1 [file cancers-17-00397-s001.zip › cancers-3414478-supplementary.pdf]

Supplementary

Table supplementary S1 Associated factors of smoking behavior transformations [aRR(95%CI)]

Previous behavior	Current behavior	Male vs Female	Younger vs Elder	high vs low education	Municipality vs County	large vs small hospital	positive vs negative OPMDs
None	None	ref	ref	ref	ref	ref	ref
	Low dose	1.1 (1.09-1.11)	1.19 (1.18-1.21)	1.22 (1.21-1.24)	1.4 (1.39-1.42)	1.65 (1.63-1.67)	0.86 (0.84-0.87)
	High dose	2.72 (2.6-2.85)	1.71 (1.67-1.75)	0.82 (0.8-0.85)	1.19 (1.16-1.22)	1.3 (1.27-1.34)	1.37 (1.32-1.43)
Low dose	None	1.26 (1.24-1.27)	0.43 (0.42-0.43)	0.68 (0.67-0.68)	0.74 (0.73-0.75)	0.98 (0.97-0.99)	0.86 (0.84-0.88)
	Low dose	ref	ref	ref	ref	ref	ref
	High dose	1.8 (1.77-1.82)	1.23 (1.22-1.25)	0.69 (0.69-0.7)	0.88 (0.88-0.89)	0.86 (0.86-0.87)	1.4 (1.37-1.42)
High dose	None	0.99 (0.94-1.03)	0.44 (0.43-0.45)	0.83 (0.81-0.85)	0.81 (0.8-0.83)	1.19 (1.16-1.21)	0.93 (0.91-0.96)
	Low dose	0.71 (0.7-0.72)	0.86 (0.85-0.87)	1.11 (1.09-1.12)	1.07 (1.06-1.08)	0.99 (0.98-1)	0.87 (0.86-0.88)
	High dose	ref	ref	ref	ref	ref	ref

Table supplementary S2 Associated factors of betel quid chewing behavior transformations [aRR(95%CI)]

Previous behavior	Current behavior	Male vs Female	Younger vs Elder	high vs low education	Municipality vs County	large vs small hospital	positive vs negative OPMDs
None	None	ref	ref	ref	ref	ref	ref
	Low dose	2.18 (2.15-2.21)	1.76 (1.74-1.78)	0.73 (0.72-0.74)	0.66 (0.66-0.67)	0.95 (0.94-0.96)	1.25 (1.23-1.27)
	High dose	2.87 (2.78-2.99)	2.15 (2.1-2.22)	0.53 (0.52-0.55)	0.65 (0.63-0.66)	0.93 (0.91-0.95)	2.02 (1.94-2.09)
Low dose	None	1.64 (1.62-1.67)	0.78 (0.77-0.79)	1.03 (1.02-1.05)	1.25 (1.24-1.26)	1.12 (1.11-1.13)	0.94 (0.92-0.95)
	Low dose	ref	ref	ref	ref	ref	ref
	High dose	1.09 (1.06-1.13)	1.21 (1.18-1.24)	0.68 (0.66-0.7)	0.86 (0.84-0.87)	0.98 (0.96-1)	1.53 (1.48-1.57)
High dose	None	1.73 (1.65-1.8)	0.61 (0.59-0.63)	1.07 (1.04-1.1)	1.24 (1.21-1.26)	1.27 (1.25-1.3)	0.94 (0.92-0.96)
	Low dose	0.7 (0.67-0.72)	0.77 (0.75-0.79)	1.09 (1.06-1.12)	1.01 (0.98-1.03)	1 (0.98-1.03)	0.91 (0.89-0.93)
	High dose	ref	ref	ref	ref	ref	ref

Table supplementary S3 Associated factors of only smoking behavior transformations [aRR(95%CI)]

Previous behavior	Current behavior	Male vs Female	Younger vs Elder	high vs low education	Municipality vs County	large vs small hospital	positive vs negative OPMDs
None	None	ref	ref	ref	ref	ref	ref
	Low dose	0.95 (0.94-0.96)	1.24 (1.22-1.25)	1.19 (1.18-1.21)	1.34 (1.33-1.36)	1.7 (1.68-1.72)	0.93 (0.9-0.95)
	High dose	2.75 (2.6-2.88)	1.76 (1.71-1.81)	0.82 (0.8-0.85)	1.2 (1.17-1.23)	1.35 (1.31-1.39)	1.5 (1.43-1.58)
Low dose	None	1.38 (1.36-1.4)	0.44 (0.44-0.45)	0.66 (0.65-0.67)	0.79 (0.79-0.8)	0.96 (0.94-0.97)	0.88 (0.85-0.9)
	Low dose	ref	ref	ref	ref	ref	ref
	High dose	1.72 (1.7-1.73)	1.18 (1.16-1.19)	0.7 (0.69-0.71)	0.92 (0.91-0.93)	0.86 (0.85-0.87)	1.33 (1.3-1.36)
High dose	None	1.35 (1.3-1.41)	0.52 (0.51-0.53)	0.8 (0.78-0.82)	0.85 (0.83-0.87)	1.13 (1.1-1.16)	1.05 (1.01-1.09)
	Low dose	0.76 (0.75-0.77)	0.91 (0.9-0.92)	1.1 (1.09-1.11)	1.08 (1.07-1.09)	0.96 (0.95-0.98)	0.86 (0.85-0.88)
	High dose	ref	ref	ref	ref	ref	ref
